# Neurofibromatosis Type 1 Presenting with Plexiform Neurofibromas in Two Patients: MRI Features

**DOI:** 10.1155/2012/498518

**Published:** 2012-09-20

**Authors:** Ahmet Mesrur Halefoglu

**Affiliations:** Department of Radiology, Sisli Etfal Training and Research Hospital, Sisli, 34360 Istanbul, Turkey

## Abstract

Neurofibromatosis type 1 (NF1), also known as peripheral neurofibromatosis or von Recklinghausen's disease, is one of the most common genetic disorders. It is inherited in an autosomal dominant pattern. Multiple cutaneous neurofibromas are hallmark lesions of NF1. Localized and plexiform neurofibromas of the paraspinal and sacral region are the most common abdominal neoplasms in NF1. Herein, we report two patients with a known history of NF1 presenting with multiple, extensive localized and plexiform neurofibromas. We describe the important distinguishing features of these tumors as seen on magnetic resonance imaging (MRI), including very bright signal intensity and target sign on T2 weighted images.

## 1. Introduction 

Neurofibromatosis, also known as von Recklinghausen's disease, is a phakomatosis that displays a wide spectrum of clinical expression with neurocutaneous abnormalities and involvement of multiple organ systems. There are two major forms, designated neurofibromatosis type 1 (NF1) and neurofibromatosis type 2 (NF2), which are clinically and genetically distinct. NF1 is commonly associated with peripheral nerve sheath tumors, whereas NF2 primarily affects the central nervous system [1]. 

NF1 is one of the most common genetic disorders affecting approximately one in 3.000 patients [2]. Mutations of the NF1 gene which is located on chromosome 17 lead to abnormal tumor suppression. Consequently, patients with NF1 have an increased prevalence of benign and malignant neoplasms. 

NF1 affects all races and both sexes equally, occurring in the population with a prevalence of approximately one in 3.000 persons. It is autosomal dominantly inherited although up to 50% of cases may evolve from a new mutation, with advanced paternal age as a risk factor [3]. The large and complex NF1 gene is located on chromosome 17 and mutations of that gene resulting with hyperplasias, hamartomas and benign and malignant neoplasms occur in a variety of tissues and organs as a result of abnormal tumor suppression.

## 2. Case Report 

**Figure 1:**
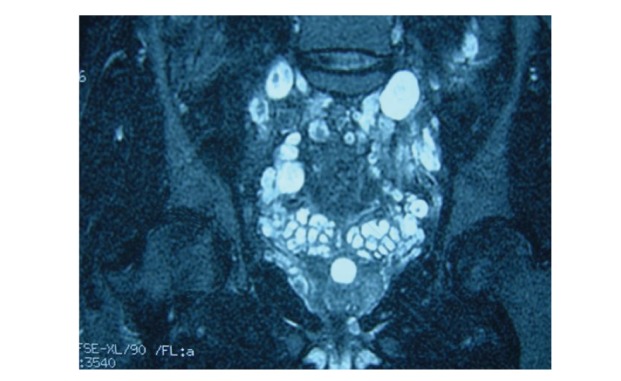
Coronal FSE fat-suppressed T2 weighted image:multiple ovoid-shaped high signal intensity neurofibromas are seen throughout the sacral and pelvic regions. Note that some neurofibromas demonstrate characteristic target sign.

**Figure 2:**
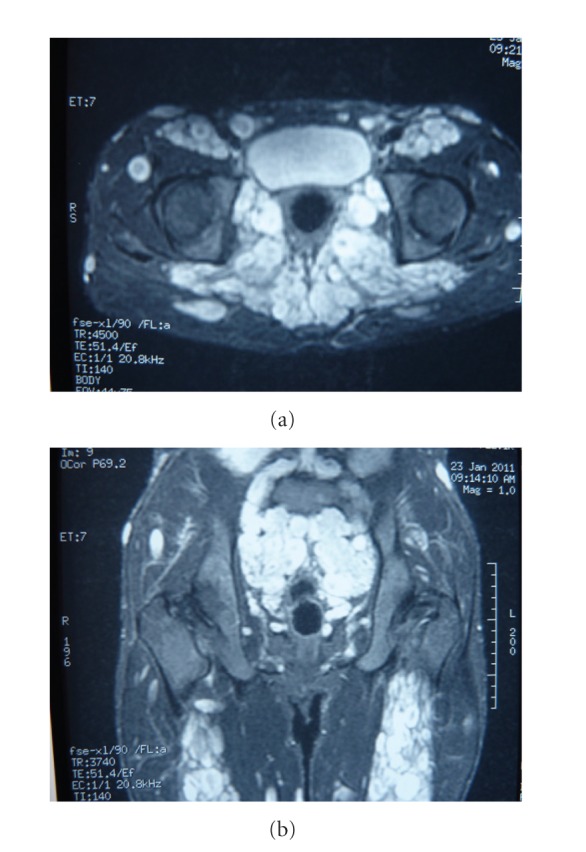
(a) Axial FSE fat-suppressed T2 weighted image shows extensive, conglomerate masses in the pelvis, gluteal region and along the bilateral sciatic nerves. (b) Coronal FSE fat-suppressed T2 weighted image of the same patient demonstrates that neurofibromas have a characteristic bright signal intensity on these images.

Two male patients, 31 and 23 years old, respectively, with a known history of NF1 were admitted to our hospital for routine control. Both patients have no current complaints. Their physical examinations and laboratory test results were found within normal limits. They underwent a routine magnetic resonance imaging (MRI) evaluation using a 1.5 tesla magnet (GE, Signa, Milwaukee, Wisconsin, USA). We performed precontrast and postcontrast (after 0.1 mmol/kg gadolinium) axial and coronal FSPGR/80 T1 weighted gradient echo and axial FSE fat-suppressed T2 weighted sequences. On these images, numerous, extensive neurofibromas localized in the sacral and pelvic regions were found showing very high signal intensity on T2 weighted images of the first patient. Some neurofibromas demonstrated a pathognomonic target sign signal intensity (Figure 1). In the second patient, again extensive conglomerate masses in the pelvis, gluteal region and along the bilateral sciatic nerves consistent with plexiform neurofibromas were detected. These lesions similarly exhibited a very bright signal intensity on T2 weighted images (Figures 2(a) and 2(b)).

Because both patients had NF1 history and biopsy of these lesions is usually reserved for cases in which the diagnosis of NF1 is in question, we did not perform this procedure. Currently, the patients are closely being followed up clinically and by MRI.

## 3. Discussion 

The diagnosis of NF1 is largely based on clinical criteria established by the National Institutes of Health Consensus Development Conference [4], that is, the presence of two or more of the following: cafe-au-lait macules or neurofibromas, Lisch nodules, axillary or inguinal freckling, optic glioma, distinctive osseous lesions or first-degree relatives with NF1. Because these clinical criteria are well established and widely accepted, pathological confirmation of neurofibroma is not a requirement and is not routinely recommended for the diagnosis of NF1. 

Neurofibromas are benign nerve sheath tumors and the hallmark lesion of the NF1. Plexiform neurofibromas are pathognomonic for NF1, usually involving a long segment of a major nerve trunk and extending into the nerve branches and they result in the so-called bag of worms appearance on gross inspection and cross-sectional imaging. Computed tomography (CT) of plexiform neurofibromas shows large multilobulated low-attenuation masses usually within a major nerve distribution. The attenuation values range from 20 to 25 HU on nonenhanced scans and 30–50 HU on intravenous contrast-enhanced scans [5]. The low attenuation of neurofibromas has been attributed to myxoid and mucinous stroma that can be observed microscopically within these tumors [6]. Tonsgard et al. [7] reported intravenous contrast enhancement in 50% of their patients with abdominal or pelvic plexiform neurofibromas. This enhancement may be homogenous or heterogenous. 

MRI reveals large conglomerate masses consisting of innumerable neurofibromas, diffusely thickening the involved nerve and often extending into nerve branches. The MRI features of neurofibromas are characteristic and can be helpful in the evaluation of a mass in a patient with known NF1. Neurofibromas show characteristically low signal intensity on T1 weighted images and heterogenous high signal intensity on T2 weighted images. The high T2 signal corresponds pathologically to areas of cystic degeneration or myxoid matrix and the low T2 signal represents collagen and fibrous tissue [8]. The areas of low T2 signal enhance following gadolinium administration. Plexiform neurofibromas have a characteristic ringlike or septated pattern that represents the complex fascicular arrangement typical of these tumors [9]. This pattern is best observed on T2 weighted images and gadolinium-enhanced T1 weighted images. Past MRI studies show that plexiform neurofibromas typically have a target-like appearance on T2 weighted MR images, with central low signal intensity and peripheral high signal intensity [8, 10]. The appearance of such a target sign may be caused by intralesional necrosis and hemorrhage. The multiplanar capability of MRI is also useful for defining the extent of plexiform neurofibromas because they may grow to large sizes and involve adjacent tissue plans and organs.
